# The Development of Brain Network in Males with Autism Spectrum Disorders from Childhood to Adolescence: Evidence from fNIRS Study

**DOI:** 10.3390/brainsci11010120

**Published:** 2021-01-18

**Authors:** Wei Cao, Huilin Zhu, Yan Li, Yu Wang, Wuxia Bai, Uchong Lao, Yingying Zhang, Yan Ji, Sailing He, Xiaobing Zou

**Affiliations:** 1 Centre for Optical and Electromagnetic Research, South China Academy of Advanced Optoelectronics, South China Normal University (SCNU), Guangzhou 510006, China; wei.cao@coer-scnu.org; 2 Child Development & Behavior Center, Third Affiliated Hospital of SUN YAT-SEN University, No.2693, Kaichuang revenue, Lingnan Campuses, Guangzhou 510080, China; zhuhlin6@mail.sysu.edu.cn (H.Z.); liyan88@mail2.sysu.edu.cn (Y.L.); wangy549@mail2.sysu.edu.cn (Y.W.); wuxia.bai@postgrad.curtin.edu.au (W.B.); laouchong@mail2.sysu.edu.cn (U.L.); zhangyy335@mail2.sysu.edu.cn (Y.Z.); jiyan196810@163.com (Y.J.)

**Keywords:** autism spectrum disorder, adolescence, functional near-infrared spectroscopy (fNIRS), graph theory, theory-of-mind network

## Abstract

In the current study, functional near-infrared spectroscopy (fNIRS) was used to collect resting-state signals from 77 males with autism spectrum disorders (ASD, age: 6~16.25) and 40 typically developing (TD) males (age: 6~16.58) in the theory-of-mind (ToM) network. The graph theory analysis was used to obtain the brain network properties in ToM network, and the multiple regression analysis demonstrated that males with ASD showed a comparable global network topology, and a similar age-related decrease in the medial prefrontal cortex area (mPFC) compared to TD individuals. Nevertheless, participants with ASD showed U-shaped trajectories of nodal metrics of right temporo-parietal junction (TPJ), and an age-related decrease in the left middle frontal gyrus (MFG), while trajectories of TD participants were opposite. The nodal metrics of the right TPJ was negatively associated with the social deficits of ASD, while the nodal metrics of the left MFG was negatively associated with the communication deficits of ASD. Current findings suggested a distinct developmental trajectory of the ToM network in males with ASD from childhood to adolescence.

## 1. Introduction

Autism spectrum disorder (ASD) is a neurodevelopmental disorder characterized by deficits in social interactions and communication, restricted interests and repetitive behaviors [1]. Despite symptoms usually presented in the early developmental period, individuals with ASD continue to experience both improvements and challenges from childhood through adolescence to adulthood. Adolescence is a transitional period towards adulthood involving massive physical, cognitive and social maturation. It is a sensitive period for brain development, especially brain regions that are involved in social cognition [2]. Picci and Scherf proposed a “Two-Hit Model” of the development of ASD, which assumed that adolescence may be an especially vulnerable period for the improvements and outcomes of ASD. They argued that the increased social demands and pubertal hormones created a secondary hit, therefore preventing individuals with ASD from transitioning into an independent life [3]. Thus, exploring the atypical brain development during this critical time window may enable a better understanding of the neural basis of ASD and its association with outcomes. The current study tried to explored the developmental trajectory of the topology in the high-functioning males with ASD from childhood to adolescence.

ASD was assumed to associate with atypical developmental trajectories of brain connectivity and brain network topology [4], though most studies focused on the early developmental years [5,6]. A study indicated that children with ASD showed enhanced within-network connectivity compared to typically developing (TD) children, while adolescents and adults with ASD exhibited similar levels of intrinsic functional connectivity as TD individuals [7]. This result suggested a non-uniform functional connectivity profile of ASD across the lifespan. Several findings supported the atypical developmental trajectories of functional connectivity from childhood to adulthood [8,9,10,11], whereas others demonstrated a normal development in ASD, with only an overall group difference [12,13]. The results remain highly heterogeneous.

Graph theory was introduced to model the large-scale brain network topology based on the concepts of “node” and “edge”, and several global and nodal network metrics [14]. For global metrics, global and local efficiency were normally used. Global efficiency refers to the average inverse shortest path length between any two nodes in the network. It is a measurement of functional integration and quantifies the capability of transferring information through a network. Local efficiency is the efficiency of information transformation within a local subgraph consisting of only the neighbors of a given node. It is a measurement of functional segregation. For nodal metrics, the nodal degree of a single node is equal to the number of links connected to that node; nodes with higher degrees show denser connections with other nodes in the network. The betweenness centrality of a node measures how many of the shortest paths between all other node pairs in the network pass through that node. A node with higher betweenness centrality is thus crucial to efficient information communication. Nodal efficiency is defined as the inverse of the average shortest distance between one node and all other nodes of the graph [15].

While a resting-state fMRI study reported reduced modularity and local network efficiency, and higher global efficiency in adolescents and adults with ASD [16], other studies found normal brain network topology of the whole brain or in the frontoparietal and default mode network in participants with ASD [17]. From the developmental perspective, a recent study found children and adolescents with ASD showed a decreased nodal degree in Broca’s area, while adults with ASD showed a decreased nodal degree in Wernicke’s area. An increased nodal degree in the left inferior parietal lobule and left middle temporal gyrus in children and adults with ASD were also reported [18]. Another study reported age-related decreased local efficiency and stable global efficiency from childhood to early adolescence in participants with ASD [19]. A large-sample study analyzed the lifespan developmental trajectory of the brain network and found a significant negative quadratic relationship between age and modularity, as well as a positive quadratic relationship between age and global efficiency in participants with ASD. This finding suggested an atypical process of the integration and segregation in participants with ASD [20]. Nevertheless, the exact brain developmental trajectories of individuals with ASD from childhood to adolescence remained uncertain.

Social demands have been found to increase sharply during adolescence, and the brain becomes sensitive to social experience, which may affect their risk-taking [21] and peer relationships [22]. The theory-of-mind (ToM) is the ability to understand other people’s emotion, attribute perspective and intentions, and is the core of social deficits in individuals with ASD [23]. The ToM network refers to a specific set of brain regions, which consist of the medial prefrontal cortex (mPFC), temporo-parietal junction (TPJ), inferior frontal gyrus (IFG), superior temporal sulcus (STS) and posterior cingulate cortex (PCC) [24]. Adolescents with ASD were found to show atypically increased functional connectivity in the ToM network [25]. A longitudinal study found that adolescents with ASD without a history of ToM deficits showed similar enhanced activation in the mPFC, PCC and lateral temporal cortex compared to adolescents who had such a history during childhood [26]. A recent study found that children with ASD showed lower functional connectivity within self- and other-referential networks compared to TD children, while adults with ASD only exhibited lower connectivity within other-referential networks, while adolescents with ASD showed similar connectivity patterns as TD adolescents [27]. These findings suggested atypical developmental patterns of the ToM network in individuals with ASD. Recently, a large autism cohort research reported no effects of age and diagnosis on neural responses during an animated shape mentalizing task. This study suggested that altered social brain activation during a mentalizing task did not form a common neural marker of ASD, though autistic individuals did show significant social deficits. They therefore highlighted the need to interrogate social brain function with other brain measures, such as connectivity and network-based approaches [28].

The “extreme male brain” theory treated ASD as an extreme pattern of male profiles with impaired empathizing and enhanced systemizing [29]. Recently, a similar age-related development of homotopic brain connectivity was reported between ASD males and TD males, in contrast to female participants, supporting the “extreme male brain” theory from a developmental perspective [30]. However, a resting-state fMRI study found that the functional connectivity of females with ASD reflected a “neural masculinization” pattern, which referred to the hyper-connectivity levels seen in TD males, whereas the hypo-connectivity observed in males with ASD reflected a “neural feminization” pattern typically seen in TD females. These findings suggested a “sexual differentiation” theory of ASD instead of the “extreme male brain” theory [31]. Using graph theory analysis, a study on lifespan did not find a significant difference between males with ASD and TD on the developmental trajectories of global efficiency and modularity in participants with ASD [20]. However, the development of nodal metrics in the brain network of the males with ASD was underexplored.

In the current study, functional near infrared spectroscopy (fNIRS) was utilized to investigate the development trajectories of the ToM network topological properties of high-functioning participants with ASD from childhood to adolescence. The fNIRS can measure the concentration change of oxy-, deoxy- and total-hemoglobin (HbO, HbR, HbT) non-invasively based on the change of light intensity, and was suggested to be suitable for the neuroimaging study of neurodevelopmental individuals [32]. Based on previous studies, we hypothesized that the topological properties of the ToM network of males with ASD may show an atypical age-related development, and these graph metrics may be associated with the severity of ASD symptoms.

## 2. Materials and Methods

### 2.1. Participants

A total of 77 males with ASD (6~16.25 year-old) and 40 TD (6~16.58 year-old) were recruited in current study. Participants with ASD were recruited through medical records of the Child Development and Behavior Center of the Third Affiliated Hospital of Sun Yat-Sen University. Both groups were matched by age and IQ, which was measured by the Wechsler Intelligence Scale for Children (WISC- IV). Symptom severity of participants with ASD was assessed by the Autism Diagnostic Observation Schedule (ADOS) [33], and the communication and social scores of the ADOS were entered into further analysis. In total, 10 participants were administered Module 4 (Fluent Speech/Adolescent/Adult), and the rest Module 3 (Fluent Speech/Child/Adolescent/). All participants had an IQ of 70 or above. Demographic information of the participants is presented in Table 1. All participants were right-handed. Parents of participants provided informed and written consent for their participation prior to the experiment.

### 2.2. Data Acquisition

A continuous-wave fNIRS instrument (FOIRE-3000, Shimadzu, Kyoto, Japan) was used to measure the spontaneous changes of hemoglobin concentration during resting-state. 15 source and 16 detector probes were attached to the forehead. The lower edge of the probes was placed along the eyebrow, and the middle line of the patch was aligned with the inion-to-nasion midsagittal line. Detector 9 was placed on Fp1 in the 10-10 system, and detectors 3 and 15 were fitted around T3 and T4, respectively. The source-detector distance was fixed at 3 cm.

**Figure 1:**
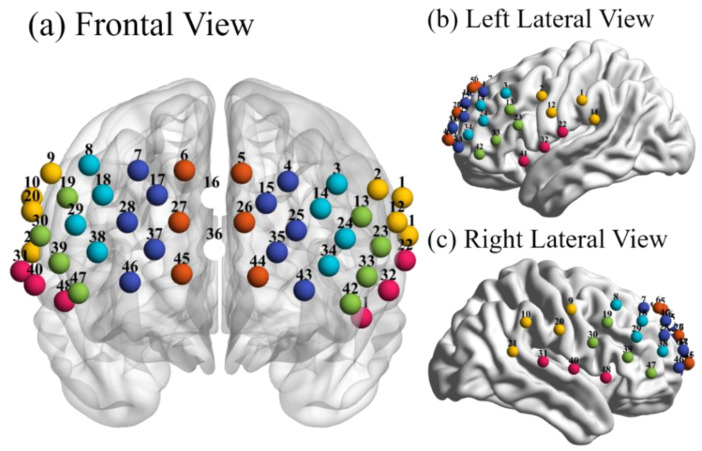
The arrangement of 48 measurement channels covering the prefrontal network of the brain: Frontal view (**a**), left and right lateral views (**b**,**c**). The anatomical positions included inferior frontal gyrus (IFG, green balls), middle frontal gyrus (MFG, light blue), superior frontal gyrus (SFG, dark blue), superior median frontal gyrus (SMFG, dark red), temporo-parietal junction (TPJ, yellow) and superior temporal gyrus (STG, pink). The figure was visualized via BrainNet Viewer [34].

The exact spatial coordinates of the optical probes and four reference points (nasion, inion, right and left ear mastoids) were obtained by a 3D digitizer (Fastrak, Polhemus, VT, USA) and then converted into Montreal Neurological Institute (MNI) space. The coordination of measurement was determined via the automated anatomical labeling (AAL) template. A total of 48 channels mainly covered the inferior frontal gyrus (IFG), middle frontal gyrus (MFG), superior frontal gyrus (SFG), superior median frontal gyrus (SMFG), temporo-parietal junction (TPJ) and superior temporal gyrus (STG) (Figure 1). The MNI coordinates and brain regions are presented in the Supplementary material (Table S1).

The near-infrared light of three wavelengths (780 nm, 805 nm, 830 nm) was used at a sampling rate of 8.7 Hz. All participants were instructed to sit in a quiet room. They were asked to close their eyes and sit still for 8 min. For those who could not sit still, an experienced pediatrician or research assistant would try to comfort them by behavioral and structural strategies. Both HbO and HbR signals were included in further analysis.

### 2.3. Data Preprocessing

First, data from the first and last 30 s was removed to avoid edge effects. Motion artifacts were corrected via spline interpolation [35] and wavelet filtering [36] using the Homer2 package in MATLAB [37]. The raw intensity was firstly transformed into the changes of optical density. Then motion artifacts were identified based on the change of raw intensity or moving standard deviation (MSD) within a certain time window against the predefined threshold. The time window was set as 1 s, and the threshold for the raw intensity and MSD were set as 0.1 and 15 as recommended by previous study [38]. The spline and wavelet methods were conducted subsequently. Approximately, 15.2 ± 3.2% signals of the ASD group and 14.8 ± 2.9% of the TD group were identified as motion artifacts, and no significant groups difference was found (*t* = 0.524, *p* = 0.601). The time series signals were then band-pass filtered between 0.009~0.08 Hz [39,40,41,42], and corrected using the 3-order polynomial detrending method. In the end, the signals were corrected against the first time point of the signals as the baseline correction.

### 2.4. Data Analysis

#### 2.4.1. Network Construction

The GRETNA toolbox was used to construct and analyze the brain network properties [43]. Pearson’s correlation coefficient between each pair of channels was computed and then transformed into *z*-values via Fisher’s *z*-transformation to improve the normality. The 48 × 48 matrix of *z*-value coefficients was then converted into a binary adjacency matrix through a series of sparsity thresholds. Only positive coefficient *z*-scores were used, because negative correlation coefficients would contaminate the retest reliability of graph theory analysis [43].

The network sparsity, defined as the ratio of the number of existing edges divided by the maximum possible number of edges in a network, could ensure the same number of edges in each sparsity threshold of both groups. Therefore, in this study, a net sparsity threshold of between 1% and 50% was adopted in increments of 1%.

#### 2.4.2. Network Analysis

To examine the topological properties of the brain network, two global metrics (i.e., global efficiency and local efficiency) were selected. The global and local efficiency of the network were calculated and normalized against the corresponding parameters of the 100 random networks, which maintained the same numbers of nodes, edges and distribution of degrees. For nodal metrics, since previous study found that betweenness centrality showed significant lower retest reliability [44], only nodal degree and nodal efficiency were utilized.

The area under the curve (AUC) of all topological metrics was also obtained to avoid the dependence on sparsity thresholds, which was the integral of the topological properties over the sparsity thresholds. Detailed information and equations of both global and nodal metrics were presented in the Supplementary material (Table S2).

#### 2.4.3. Statistical Analysis

To address the difference of the developmental trajectory of the brain network between ASD and TD participants, a multiple regression model was selected to model the relationship between age and the brain network properties. The diagnostic group, age (centered), age-squared, an age and diagnosis interaction, and an age-squared and diagnosis interaction terms were entered into the model as independent variables, and the global and nodal topological metrics of each channel of each participant as the dependent variables. To reduce multicollinearity, the age-squared term was calculated as the square of the centered age variable. The regression model was as follows:

(1) $$Y=\beta_{0}+\beta_{1}\times group+\beta_{2}\times age+\beta_{3}\times age^{2}+\beta_{4}\times age:group+\beta_{5}\times age^{2}:group+e$$

The brain-behavior relationship was evaluated with partial correlation coefficients between the network properties and the communication and social scores of ADOS, after controlling the effect of age. The false discovery rate (FDR) correction (*p* < 0.05) was adopted to adjust the multiple comparisons above [45].

## 3. Results

### 3.1. The Difference in the Development of Topology in the ToM Network

**Figure 2:**
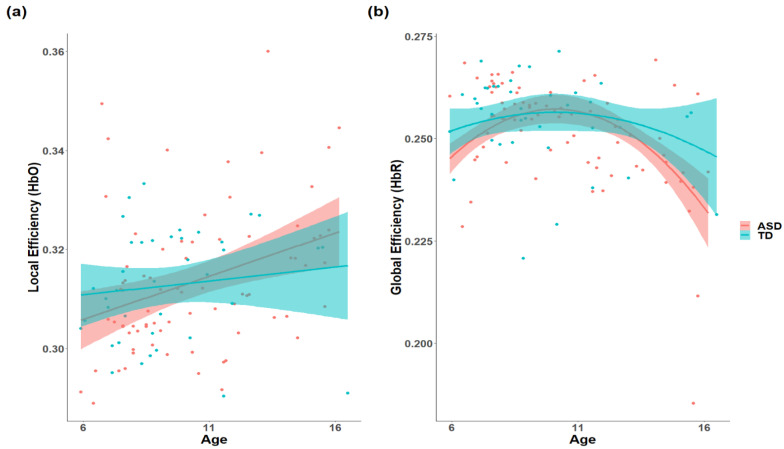
The significant main effect of age on network efficiency. The local efficiency of oxy-hemoglobin (HbO) signals (**a**) showed a positive age effect, while the global efficiency derived from deoxy-hemoglobin (HbR) signals (**b**) showed a significant effect of age-squared.

Contrary to the hypothesis, there were no significant group differences or interactions between age or age-squared and diagnosis in global and local efficiency (*p* > 0.05, FDR-corrected). The HbO signals only revealed a significant age effect on local efficiency (*R-squared* = 0.124, standardized β = 0.271, *p* = 0.050, Figure 2b), while for the HbR signals, there were significant main effects of age and age-squared in global efficiency (*R-squared* = 0.235, β = −0.257, *p* = 0.047; β = −0.343, *p* = 0.009, Figure 2a). Detailed information can be seen in Table S3.

**Figure 3:**
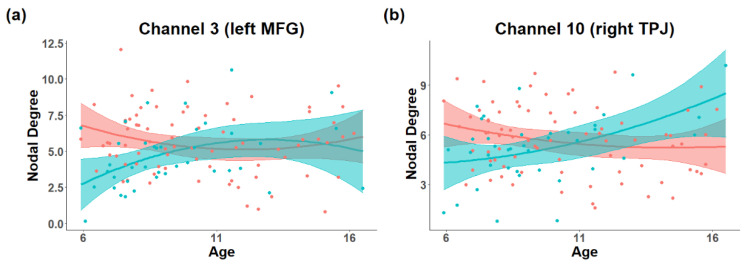

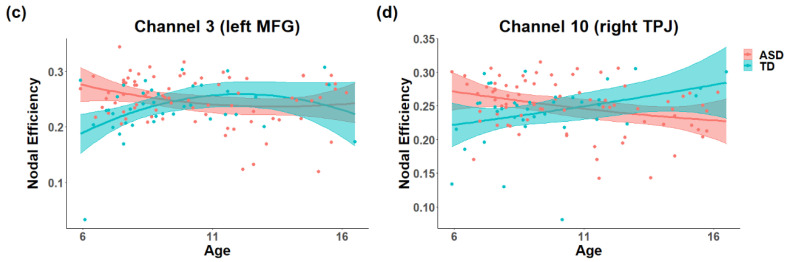
The plotted marginal effect curves of nodal metrics and age. TD participants showed an inverted U-shaped trajectory of the nodal degree (**a**) and nodal efficiency (**c**) in channel 3 in the left MFG, while participants with ASD showed U-shaped patterns. Furthermore, participants with ASD showed an age-related decrease in nodal degree (**b**) and nodal efficiency (**d**) in channel 10 in the right TPJ while the trajectory of TD was opposite.

**Figure 4:**
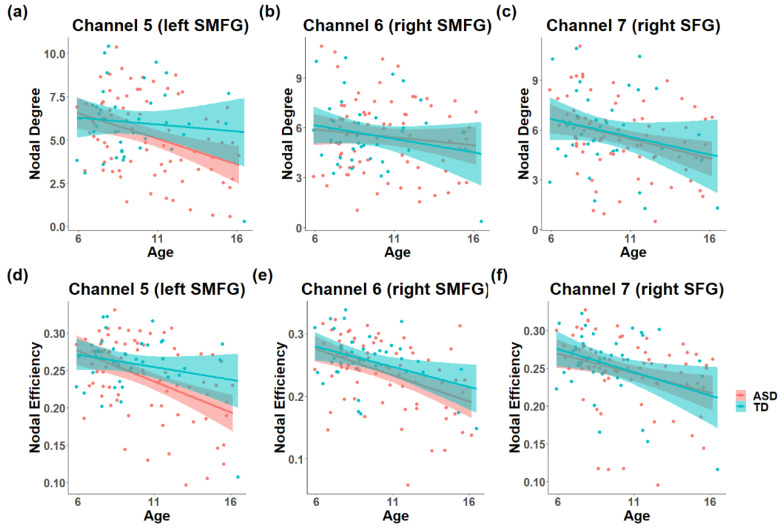
The significant main effect of age on nodal metrics. Participants showed a negative effect of age on the nodal degree of channel 5 in the left SMFG (**a**), channel 6 in the right SMFG (**b**), channel 7 in the right SFG (**c**), and nodal efficiency of channel 5 (**d**), 6 (**e**), 7 (**f**). Both nodal metrics showed an age-related decrease trajectory.

The multiple regression model revealed marginal interaction between age-squared and diagnosis in the nodal degree of channel 3 in the left MFG (HbO, *R-squared* = 0.112, β = −0.291, *p* = 0.054, Figure 3a), and channel 7 in the right SFG (HbO, *R-squared* = 0.121, β = −0.296, *p* = 0.049) showed a significant interaction between age-squared and diagnosis. TD participants showed an inverted U-shaped trajectory, while participants with ASD showed a U-shaped trajectory. Meanwhile channel 10 in the right TPJ (HbR, *R-squared* = 0.115, β = 0.376, *p* = 0.003, Figure 3b) and channel 23 in the left IFG (HbR, *R-squared* = 0.161, β = −0.375, *p* = 0.003) showed a significant interaction between age and diagnosis. Participants with ASD showed an age-related decrease in channel 10, and an age-related increase in channel 23. In addition, channel 5 in left of the SMFG (HbR, *R-squared* = 0.151, β = −0.302, *p* = 0.027, Figure 4a), channel 6 in the bilateral SMFG (HbR, *R-squared* = 0.137, β = −0.398, *p* = 0.004, Figure 4b), channel 7 in the right SFG (HbO, *R-squared* = 0.121, β = −0.447, *p* = 0.001, Figure 4c), channel 22 in the left STG (HbR, *R-squared* = 0.109, β = 0.372, *p* = 0.008) and channel 40 in the right STG (HbR, *R-squared* = 0.109, β = 0.352, *p* = 0.012) showed significant main effect of age. The nodal degree of channel 44 in the left SMFG (HbO, *R-squared* = 0.122, β = −0.284, *p* = 0.029) showed a significant main effect of diagnosis, and participants with ASD showed a significant higher nodal degree than TD. Detailed information can be seen in Table S4.

For nodal efficiency, channel 3 in the left MFG (HbO, *R-squared* = 0.125, β = −0.346, *p* = 0.021, Figure 3c) showed a significant interaction between age-squared and diagnosis. An inverted U-shaped trajectory was found in TD participants, while participants with ASD showed a U-shaped pattern. Furthermore, channel 10 in the right TPJ (HbR, *R-squared* = 0.113, β = 0.380, *p* = 0.003, Figure 3d) showed a significant interaction between age and diagnosis. Participants with ASD showed an age-related decrease while the trajectory of TD was opposite. Channel 4 in the left SFG (HbR, *R-squared* = 0.106, β = −0.334, *p* = 0.017), channel 5 in the left SMFG (HbR, *R-squared* = 0.230, β = −0.398, *p* = 0.002, Figure 4d), channel 6 in the right SMFG (HbR, *R-squared* = 0.222, β = −0.432, *p* = 0.001, Figure 4e), channel 7 in the right SFG (HbO & HbR, *R-squared* = 0.137, *B* = −0.430, *p* = 0.002; *R-squared* = 0.106, β = −0.460, *p* < 0.001, Figure 4f), channel 17 in the right SFG (HbR, *R-squared* = 0.133, β = −0.274, *p* = 0.047) and channel 18 in the right MFG (HbO, *R-squared* = 0.100, β = −0.320, *p* = 0.023) showed significant main effects of age. Channel 42 in the left IFG (HbR, *R-squared* = 0.107, β = 0.262, *p* = 0.045) showed a significant diagnosis effect, and ASD showed reduced nodal efficiency than TD. Detailed information can be seen in Table S5.

### 3.2. Associations between Topological Metrics and Severity of ASD Symptoms

**Figure 5:**
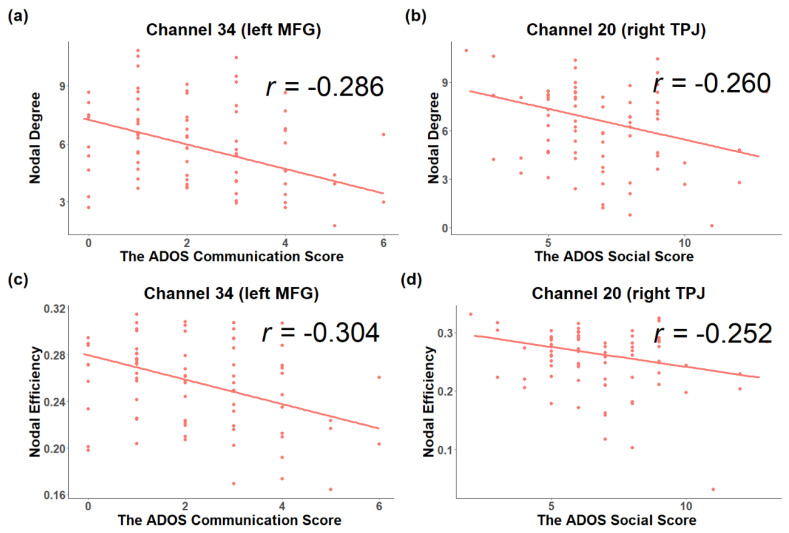
The partial correlation coefficients between nodal metrics and ADOS scores after controlling the effect of age. The nodal degree (**a**) and nodal efficiency (**c**) of the channel 34 in the left MFG showed a negative association with ADOS communication scores, while nodal degree (**b**) and nodal efficiency (**d**) of channel 41 in the left STG showed a negative association with ADOS social scores.

The partial correlation did not find any significant associations between global or local efficiency and ADOS severity. For nodal degree, channel 34 in the left MFG (HbR, *r* = −0.286, *p* = 0.012, Figure 5a) was negatively associated with ADOS communication scores, while channel 20 in the right TPJ (HbR, *r* = −0.260, *p* = 0.024, Figure 5b) and channel 41 in the left STG (HbO, *r* = −0.236, *p* = 0.040) showed negative associations with ADOS social scores. For nodal efficiency, channel 1 in the left TPJ (HbO, *r* = 0.264, *p* = 0.021) showed a positive association with ADOS social scores, while channel 20 in the right TPJ (HbR, *r* = −0.252, *p* = 0.028, Figure 5d) was negatively associated with ADOS social scores. Furthermore, channel 34 in the left MFG (HbR, *r* = −0.304, *p* = 0.008, Figure 5c) was negatively associated with ADOS communication score, while channel 48 in the right STG (HbR, *r* = 0.247, *p* = 0.031) showed a positive association with ADOS communication scores.

## 4. Discussion

In this study, we investigated the developmental trajectories of the ToM network in males with ASD from childhood to adolescence. Based on our hypothesis, we assumed that males with ASD may show atypical development of the topological properties in the ToM network. While the multiple regression model did not find any significant interaction between age or age-squared and diagnosis in global and local efficiency, the nodal metrics in bilateral SFG and SMFG only showed a significant age effect. Current results only showed significant interactions between age and/or age-squared and diagnosis in some specific regions related to ToM, which were predominantly located in the right TPJ and left MFG. The partial correlation demonstrated negative association between nodal metrics in left MFG and ADOS communication scores, and between nodal efficiency in right TPJ and ADOS social scores. These findings may provide new evidences for atypical developmental trajectories in the ToM network of ASD from childhood to adolescence.

Though previous studies have reported atypical development of global and local efficiency of children and adolescents with ASD [18,19,46], neither significant interactions between age and/or age-squared and diagnosis in global and local efficiency nor significant main effect of diagnosis were identified. Though one study found no significant age effect of functional connectivity and network topologies in the ToM network from childhood to adolescence during a social evaluation task, it was suggested that the organization of the social brain was largely set by childhood and did not undergo a large development during adolescence [47]. Current results seemed to suggest an intact developmental trajectory of the network topology in males with ASD, as a previous study also reported intact functional connectivity profiles between high-functioning ASD and TD [48]. Furthermore, we did not identify any significant associations between global and local efficiency and the severity of ASD’s core symptoms. This may suggest that atypical global and local efficiency may not be a specific feature of individuals with ASD, rather than a domain-general deficit of neurodevelopmental disorders [49,50,51,52].

Current results also imply that participants with ASD showed atypical trajectories in the right TPJ, with similar development in areas near the mPFC. The TPJ and mPFC regions were among core areas of the ToM network, and may interact as the hubs that integrated the information about the complex and dynamic social context from multiple brain networks [53]. Though the exact role of the TPJ and mPFC in the ToM network remain debatable, the TPJ, especially the right TPJ was found to be involved in various mentalizing tasks [54]. Previous task-based studies have confirmed both reduced and enhanced activation in the TPJ during various mentalizing tasks in adolescents with ASD [55,56], and the aberrant TPJ function during mentalizing tasks was associated with social impairment in ASD [57]. A causal inference study only reported a reduced brain response and decreased connectivity in the right TPJ but not mPFC in male adults with ASD [58]. Thus, current findings may imply that atypical development of the right TPJ mediated the social deficits in males with ASD from childhood to adolescence.

An fMRI study has demonstrated that only adolescents showed additional activation in the mPFC during mentalizing tasks, while the TPJ was activated in both adolescents and adults [59]. This finding suggested a shift from anterior (prefrontal cortex) regions to posterior (temporal cortex) regions within the ToM network [2]. The current study revealed a similar “anterior to posterior” pattern, as nodal metrics of the mPFC decreased, and the right TPJ increased with age in TD participants. However, no significant diagnosis effect was reported in the mPFC. Neuroimaging studies have proposed that the mPFC may be related to domain-general rather than ToM-specific functions, while the right TPJ was constantly activated during mentalizing tasks [60,61,62]. Therefore, nodal metrics did not show any significant associations with ASD symptoms. On the other hand, the negative association between nodal efficiency in the right TPJ and ADOS social score enhanced the idea that the atypical development of the right TPJ may underlie the social deficits of ASD.

A previous study reported an increased nodal degree in the left MFG in children with ASD [63]. Another study also found increased functional connectivity in the left MFG in adults with ASD [64]. Current findings extended these results, and revealed a U-shaped developmental trajectory in participants with ASD from childhood to adolescence. This result is consistent with previous findings and seems to suggest adolescence as a unique developmental period for ASD. The left MFG was found to be involved in speech production [65] and the executive control network [66], so the negative association between the nodal metrics of left MFG and ADOS communication score suggested left MFG as an important brain region associated with the communication of ASD.

## 5. Conclusions

In summary, the current study suggests an intact development of the network efficiency in the ToM network, and in the nodal metrics of the left MFG of high functioning male children and adolescent with ASD. Furthermore, the present study emphasizes that the atypical development of the right TPJ may be critical to the social deficits in the individuals with ASD from childhood to adolescence. It also indicates the importance to explore the brain development of ASD before and during continuing adolescence. This study can also act as a starting point for the future developmental studies regarding gender effect of ASD.

## Figures and Tables

**Table 1:** Demographic data.

	ASD (*n* = 77)	TD (*n* = 40)	*t*	*p*
Age (years)	10.51 (2.86) 6~16.25	9.54(2.58) 6~16.58	1.780	0.078
VIQ	105.79 (17.44)	106.05 (13.72)	−0.081	0.936
PIQ	104.68 (14.36)	108.85 (12.66)	−1.536	0.127
FSIQ	101.47 (14.85)	105.10 (12.10)	−1.322	0.189
ADOS communication	2.21 (1.50)			
ADOS social	6.79 (2.15)			

Note: ASD = autism spectrum disorders, TD = typically developing, CSS = Autism Diagnostic Observation Schedule (ADOS) calibrated severity score, VIQ, PIQ, FSIQ = verbal, performance and full-scale IQ of the Wechsler Intelligence Scale for Children (WISC), ADOS communication and social = the communication and social scores of the ADOS.

## Data Availability

The data in the present study will be available under reasonable request from the corresponding author and must be subject to the privacy restrictions and Regulations on Human Genetic Resources Management of China.

## Supplementary Materials

The following are available online at https://www.mdpi.com/2076-3425/11/1/120/s1, Table S1: The MNI coordinates and anatomical labels corresponding to the measurement channels, Table S2: The formula of topological properties, Table S3: The results of the multiple regression model on the global and local efficiency, Table S4: The results of the multiple regression model on the nodal degree of each channel, Table S5: The results of the multiple regression model on the nodal efficiency of each channel.
