# Supplementary material for: The Development of Brain Network in Males with Autism Spectrum Disorders from Childhood to Adolescence: Evidence from fNIRS Study
Source: Brain Sci. 2021 Jan 18;11(1):120. doi: 10.3390/brainsci11010120 (PMC7830916; doi:10.3390/brainsci11010120)
Supplement: Supplementary file 1 [file brainsci-11-00120-s001.pdf]

Supplementary Materials

Table S1. The MNI coordinates and anatomical labels corresponding to the measurement channels.

Channel	MNI			Brain Regions	ROIs
	x	y	z		
1	−67	−23	36	SupraMarginal_L	TPJ
2	−58	6	39	SupraMarginal_L	TPJ
3	−43	32	41	Frontal_Mid_L	MFG
4	−25	48	42	Frontal_Sup_L	SFG
5	−8	56	45	Frontal_Sup_Medial_L	SMFG
6	13	53	46	Frontal_Sup_Medial_R	SMFG
7	30	43	46	Frontal_Sup_R	SFG
8	48	24	48	Frontal_Mid_R	MFG
9	62	−7	45	SupraMarginal_R	TPJ
10	69	−38	36	SupraMarginal_R	TPJ
11	−69	−32	22	SupraMarginal_L	TPJ
12	−65	−1	27	SupraMarginal_L	TPJ
13	−52	29	29	Frontal_Inf_Tri_L	IFG
14	−37	50	32	Frontal_Mid_L	MFG
15	−17	61	34	Frontal_Sup_L	SFG
16	3	60	36	Frontal_Sup_Medial_L	0
17	23	58	37	Frontal_Sup_R	SFG
18	43	42	37	Frontal_Mid_R	MFG
19	56	18	36	Frontal_Inf_Oper_R	IFG
20	69	−15	31	SupraMarginal_R	TPJ
21	69	−47	16	SupraMarginal_R	TPJ
22	−68	−9	13	Temporal_Sup_R	STG
23	−59	23	18	Frontal_Inf_Tri_L	IFG
24	−46	48	21	Frontal_Mid_L	MFG
25	−28	62	24	Frontal_Sup_L	SFG

26	−9	67	27	Frontal_Sup_Medial_L	SMFG
27	15	67	27	Frontal_Sup_Medial_R	SMFG
28	34	57	27	Frontal_Sup_R	SFG
29	53	38	26	Frontal_Mid_R	MFG
30	66	8	22	Frontal_Inf_Tri_R	IFG
31	73	−26	9	Temporal_Sup_R	STG
32	−62	4	2	Temporal_Sup_L	STG
33	−54	39	7	Frontal_Inf_Tri_L	IFG
34	−40	59	11	Frontal_Mid_L	MFG
35	−21	70	16	Frontal_Sup_L	SFG
36	2	67	17	Frontal_Sup_Medial_L	0
37	24	70	17	Frontal_Sup_R	SFG
38	45	56	16	Frontal_Mid_R	MFG
39	59	32	12	Frontal_Inf_Tri_R	IFG
40	68	−5	4	Temporal_Sup_R	STG
41	−52	19	−8	Temporal_Pole_Sup_L	STG
42	−48	51	−3	Frontal_Inf_Tri_L	IFG
43	−31	66	2	Frontal_Sup_L	SFG
44	−14	73	7	Frontal_Sup_Medial_L	SMFG
45	14	74	8	Frontal_Sup_Medial_R	SMFG
46	33	67	5	Frontal_Sup_R	SFG
47	52	48	1	Frontal_Mid_R	IFG
48	57	17	−2	Temporal_Pole_Sup_R	STG

IFG = the inferior frontal gyrus, MFG = middle frontal gyrus, SFG= superior median frontal gyrus, SMFG= superior median frontal gyrus, TPJ = temporo-parietal junction, STG= superior temporal gyrus. The brain regions were labeled after Anatomic anatomical labeling (AAL).

Table S2. The formula of topological properties.

	Formula	Comments
Global efficiency	$Eg = \frac{1}{N} E_i = \frac{1}{N} \sum_{i \in N} \frac{\sum_{j \in N, j \neq i} d_{ij}}{N-1}$	N is the number of nodes in network, E_i is the efficiency of nodal i, d_{ij} is the shortest path length between node i and j.
Local efficiency	$Eloc = \frac{1}{N} \sum_{i \in G} \frac{\sum [d_{jh}(N_i)]^{-1}}{k_i (k_i - 1)}$	d_{jh} is the shortest path length between node j and h that contains only neighbors of i, k_i is the degree of node i.
Nodal degree	$K_i = \sum_{j \in G} a_{ij}$	a_{ij} is the connectivity status after converted into binarized network.
Nodal efficiency	$E_i = \frac{\sum_{j \in N, j \neq i} d_{ij}}{N-1}$	d_{ij} is the shortest path length between node i and j

Table S3. The results of the multiple regression model on the global and local efficiency.

Channel		Global Efficiency	Local Efficiency
Diagnosis	HbO	0.008 (0.949)	−0.025 (0.837)
	HbR	0.173 (0.179)	0.058 (0.656)
Age	HbO	−0.256 (0.057)	−0.257 (0.047) *
	HbR	0.271 (0.050) *	0.142 (0.311)
Age^2	HbO	−0.218 (0.108)	−0.343 (0.009) *
	HbR	0.150 (0.278)	0.141 (0.319)
Diagnosis: age	HbO	−0.014 (0.905)	0.034 (0.773)
	HbR	−0.021 (0.868)	0.127 (0.318)

Diagnosis: age²	HbO	0.087 (0.547)	0.150 (0.281)
	HbR	−0.279 (0.062)	−0.063 (0.679)

*: $p < 0.05$. Note: the number outside the bracket was the standardized β value, and the number in the bracket was the *p*-value.

Table S4. The results of the multiple regression model on the nodal degree of each channel.

Channel	Brain Regions	Diagnosis		Age		Age²		Diagnosis : Age		Diagnosis : Age²	
		HbO	HbR	HbO	HbR	HbO	HbR	HbO	HbR	HbO	HbR
1	Left TPJ	0.075	−0.123	−0.239	−0.020	0.275	0.136	0.085	−0.152	0.013	−0.018
		(0.575)	(0.359)	(0.094)	(0.891)	(0.056)	(0.347)	(0.510)	(0.243)	(0.934)	(0.908)
2	Left TPJ	0.122	−0.190	−0.179	0.073	0.295	−0.094	0.020	0.014	−0.074	0.259
		(0.361)	(0.160)	(0.210)	(0.613)	(0.042)	(0.517)	(0.876)	(0.912)	(0.634)	(0.098)
3	Left	0.003	0.048	−0.216	−0.110	0.181	−0.127	0.359	0.199	−0.291	−0.205
	MFG	(0.984)	(0.714)	(0.120)	(0.430)	(0.195)	(0.367)	(0.005) *	(0.120)	(0.054)	(0.180)
4	Left SFG	0.188	0.100	−0.150	−0.265	0.089	0.028	0.108	0.172	−0.221	−0.129
		(0.167)	(0.456)	(0.301)	(0.065)	(0.542)	(0.848)	(0.413)	(0.187)	(0.160)	(0.407)
5	Left	0.051	0.207	0.010	−0.302	−0.120	−0.109	−0.155	0.178	0.153	−0.122
	SMFG	(0.704)	(0.103)	(0.943)	(0.027) *	(0.403)	(0.422)	(0.234)	(0.149)	(0.322)	(0.406)
6	Right	0.026	0.215	−0.078	−0.398	−0.072	0.095	−0.037	0.105	−0.050	−0.173
	SMFG	(0.845)	(0.094)	(0.588)	(0.004) *	(0.62)	(0.489)	(0.775)	(0.396)	(0.747)	(0.242)
7	Right	0.198	0.164	−0.447	−0.367	0.242	0.169	0.144	0.179	−0.296	−0.083
	SFG	(0.127)	(0.216)	(0.001)	(0.010) *	(0.083)	(0.236)	(0.251)	(0.163)	(0.049) *	(0.589)
8	Right	0.006	−0.030	−0.071	−0.182	−0.069	0.102	0.014	0.204	−0.101	−0.174
	MFG	(0.963)	(0.823)	(0.623)	(0.204)	(0.635)	(0.48)	(0.917)	(0.120)	(0.521)	(0.263)

9	Right	0.179	−0.251	−0.020	−0.086	0.033	0.003	0.109	−0.004	−0.293	0.131
	TPJ	(0.184)	(0.064)	(0.889)	(0.548)	(0.818)	(0.984)	(0.403)	(0.975)	(0.062)	(0.402)
10	Right	0.022	−0.077	0.226	−0.237	−0.151	0.09	−0.041	0.376	0.077	0.018
	TPJ	(0.869)	(0.551)	(0.119)	(0.088)	(0.301)	(0.516)	(0.757)	(0.003) *	(0.624)	(0.905)
11	Left TPJ	0.060	−0.068	0.073	0.291	−0.032	−0.173	−0.031	−0.086	−0.013	0.104
		(0.663)	(0.613)	(0.618)	(0.045)	(0.827)	(0.232)	(0.814)	(0.508)	(0.933)	(0.505)
12	Left TPJ	−0.017	−0.120	0.067	0.088	0.044	0.010	−0.064	0.062	−0.059	0.033
		(0.902)	(0.372)	(0.648)	(0.540)	(0.765)	(0.947)	(0.630)	(0.634)	(0.710)	(0.834)
13	Left IFG	0.052	−0.076	−0.024	0.175	0.058	0.040	−0.01	−0.153	0.102	0.197
		(0.702)	(0.571)	(0.869)	(0.22)	(0.688)	(0.782)	(0.941)	(0.239)	(0.515)	(0.203)
14	Left	0.144	0.068	−0.231	−0.066	0.180	0.063	0.411	0.065	−0.210	−0.058
	MFG	(0.274)	(0.618)	(0.100)	(0.654)	(0.201)	(0.669)	(0.002)	(0.627)	(0.167)	(0.714)
15	Left SFG	−0.064	0.100	0.099	−0.111	−0.157	−0.026	−0.109	−0.149	−0.066	−0.078
		(0.634)	(0.449)	(0.491)	(0.431)	(0.278)	(0.853)	(0.402)	(0.243)	(0.672)	(0.610)
16	Left	−0.128	−0.051	−0.067	−0.232	−0.080	−0.062	0.032	0.034	−0.034	−0.005
	SMFG	(0.342)	(0.700)	(0.639)	(0.103)	(0.58)	(0.666)	(0.805)	(0.789)	(0.828)	(0.972)
17	Right	0.180	0.138	−0.188	−0.182	0.106	−0.009	−0.017	0.027	−0.039	−0.127
	SFG	(0.178)	(0.302)	(0.187)	(0.202)	(0.458)	(0.951)	(0.896)	(0.836)	(0.801)	(0.412)
18	Right	0.083	−0.138	−0.261	−0.017	0.074	−0.141	0.051	−0.004	−0.083	0.184
	MFG	(0.537)	(0.309)	(0.070)	(0.907)	(0.609)	(0.336)	(0.696)	(0.978)	(0.590)	(0.243)
19	Right	−0.025	0.064	0.109	−0.306	−0.251	0.321	−0.124	0.024	0.061	−0.085
	IFG	(0.852)	(0.628)	(0.445)	(0.032)	(0.084)	(0.026)	(0.342)	(0.851)	(0.695)	(0.578)
20	Right	0.045	−0.166	−0.337	0.058	0.178	−0.034	0.129	−0.098	−0.117	0.039
	TPJ	(0.734)	(0.221)	(0.020)	(0.689)	(0.218)	(0.817)	(0.321)	(0.457)	(0.449)	(0.802)
21	Right	0.136	−0.025	−0.257	0.003	0.065	−0.085	0.277	0.020	−0.224	0.074
	TPJ	(0.310)	(0.853)	(0.073)	(0.984)	(0.651)	(0.563)	(0.034)	(0.883)	(0.147)	(0.642)

22	Right STG	−0.097 (0.473)	0.057 (0.662)	−0.078 (0.588)	0.372 (0.008) *	0.155 (0.289)	<0.001 (1.000)	0.004 (0.978)	−0.079 (0.532)	0.013 (0.936)	−0.067 (0.656)
23	Left IFG	−0.089 (0.501)	−0.102 (0.419)	0.188 (0.184)	0.210 (0.120)	0.023 (0.871)	0.169 (0.213)	−0.199 (0.123)	−0.375 (0.003) *	0.249 (0.105)	0.199 (0.173)
24	Left MFG	−0.091 (0.495)	−0.097 (0.472)	−0.071 (0.622)	0.157 (0.277)	0.097 (0.500)	0.020 (0.892)	0.012 (0.923)	−0.195 (0.138)	0.194 (0.213)	0.122 (0.437)
25	Left SFG	−0.237 (0.078)	−0.057 (0.669)	0.157 (0.273)	−0.166 (0.243)	−0.145 (0.315)	−0.073 (0.608)	−0.034 (0.794)	0.235 (0.071)	0.086 (0.579)	−0.073 (0.636)
26	Left SMFG	−0.150 (0.272)	−0.031 (0.817)	−0.008 (0.954)	−0.096 (0.506)	−0.031 (0.831)	−0.064 (0.66)	−0.012 (0.928)	0.096 (0.464)	0.072 (0.650)	0.151 (0.334)
27	Right SMFG	0.138 (0.308)	−0.071 (0.598)	0.135 (0.349)	−0.128 (0.371)	−0.057 (0.693)	−0.047 (0.743)	−0.045 (0.732)	0.143 (0.274)	−0.138 (0.377)	−0.105 (0.497)
28	Right SFG	−0.096 (0.474)	−0.164 (0.222)	−0.257 (0.076)	−0.088 (0.537)	0.082 (0.573)	−0.147 (0.308)	0.111 (0.393)	0.116 (0.373)	−0.004 (0.977)	0.162 (0.296)
29	Right MFG	−0.254 (0.060)	−0.194 (0.145)	0.077 (0.591)	0.235 (0.098)	−0.118 (0.414)	−0.062 (0.661)	−0.194 (0.136)	−0.052 (0.687)	0.112 (0.470)	0.059 (0.699)
30	Right IFG	−0.007 (0.958)	−0.375 (0.003)	0.143 (0.323)	−0.195 (0.138)	−0.203 (0.164)	0.235 (0.071)	0.031 (0.815)	0.096 (0.464)	0.135 (0.389)	0.143 (0.274)
31	Right STG	0.101 (0.460)	−0.002 (0.988)	−0.087 (0.551)	0.031 (0.830)	0.096 (0.516)	0.123 (0.402)	0.050 (0.707)	0.009 (0.944)	−0.077 (0.628)	−0.081 (0.606)
32	Left STG	0.120 (0.366)	0.112 (0.393)	0.077 (0.587)	0.140 (0.319)	−0.047 (0.741)	0.211 (0.137)	0.124 (0.335)	0.023 (0.859)	0.124 (0.418)	−0.071 (0.640)
33	Left IFG	0.105 (0.438)	0.332 (0.013)	−0.009 (0.953)	0.096 (0.497)	0.026 (0.861)	−0.002 (0.988)	0.082 (0.536)	0.110 (0.393)	0.032 (0.841)	−0.157 (0.307)
34	Left MFG	−0.083 (0.543)	−0.067 (0.620)	0.033 (0.818)	−0.082 (0.568)	−0.065 (0.660)	0.129 (0.370)	0.042 (0.752)	−0.176 (0.177)	0.119 (0.451)	0.063 (0.686)

35	Left SFG	−0.075<br>(0.571)	−0.103<br>(0.443)	−0.159<br>(0.264)	−0.007<br>(0.961)	0.022<br>(0.877)	−0.173<br>(0.231)	−0.152<br>(0.237)	−0.158<br>(0.227)	−0.004<br>(0.977)	0.193<br>(0.216)
36	Left SMFG	−0.061<br>(0.651)	−0.29<br>(0.032)	0.148<br>(0.307)	0.117<br>(0.414)	−0.119<br>(0.414)	−0.131<br>(0.366)	0.067<br>(0.612)	−0.123<br>(0.345)	0.086<br>(0.581)	0.207<br>(0.184)
37	Right SFG	−0.180<br>(0.176)	−0.164<br>(0.216)	−0.153<br>(0.279)	−0.173<br>(0.223)	0.157<br>(0.271)	0.161<br>(0.258)	−0.154<br>(0.232)	−0.055<br>(0.671)	0.036<br>(0.815)	0.199<br>(0.196)
38	Right MFG	0.034<br>(0.801)	0.046<br>(0.733)	0.006<br>(0.968)	0.048<br>(0.742)	0.144<br>(0.321)	−0.079<br>(0.590)	0.088<br>(0.502)	−0.115<br>(0.383)	−0.183<br>(0.242)	−0.025<br>(0.873)
39	Right IFG	−0.078<br>(0.566)	0.051<br>(0.701)	0.150<br>(0.303)	0.281<br>(0.051)	−0.125<br>(0.393)	−0.008<br>(0.958)	−0.099<br>(0.454)	−0.163<br>(0.208)	0.207<br>(0.190)	0.008<br>(0.957)
40	Right STG	0.059<br>(0.656)	−0.003<br>(0.979)	0.310<br>(0.029)	0.352<br>(0.012)	−0.136<br>(0.338)	0.022<br>(0.875)	−0.010<br>(0.939)	−0.100<br>(0.429)	0.097<br>(0.526)	0.056<br>(0.711)
41	Left STG	0.075<br>(0.566)	0.241<br>(0.075)	0.261<br>(0.062)	0.028<br>(0.845)	−0.255<br>(0.071)	−0.028<br>(0.846)	0.119<br>(0.347)	0.128<br>(0.328)	0.106<br>(0.484)	−0.139<br>(0.371)
42	Left IFG	0.112<br>(0.399)	0.237<br>(0.072)	0.297<br>(0.038)	0.257<br>(0.068)	−0.211<br>(0.142)	−0.133<br>(0.345)	−0.087<br>(0.501)	0.089<br>(0.485)	0.098<br>(0.524)	−0.166<br>(0.273)
43	Left SFG	−0.190<br>(0.155)	0.019<br>(0.891)	0.056<br>(0.691)	0.004<br>(0.980)	0.005<br>(0.973)	−0.067<br>(0.646)	−0.225<br>(0.083)	−0.094<br>(0.477)	−0.003<br>(0.984)	−0.058<br>(0.711)
44	Left SMFG	−0.284<br>(0.029) *	0.041<br>(0.762)	0.266<br>(0.055)	−0.247<br>(0.088)	0.044<br>(0.752)	0.115<br>(0.427)	−0.241<br>(0.056)	0.201<br>(0.126)	0.215<br>(0.151)	−0.160<br>(0.307)
45	Right SMFG	−0.046<br>(0.734)	0.165<br>(0.224)	0.102<br>(0.483)	0.084<br>(0.563)	−0.012<br>(0.936)	0.037<br>(0.797)	0.007<br>(0.956)	−0.032<br>(0.805)	−0.028<br>(0.859)	−0.034<br>(0.828)
46	Right SFG	0.020<br>(0.879)	0.044<br>(0.750)	0.188<br>(0.190)	0.036<br>(0.807)	−0.115<br>(0.423)	−0.034<br>(0.819)	−0.243<br>(0.063)	−0.025<br>(0.853)	0.169<br>(0.278)	−0.074<br>(0.639)
47	Right MFG	−0.093<br>(0.485)	0.062<br>(0.639)	0.336<br>(0.019)	0.270<br>(0.060)	−0.122<br>(0.394)	0.011<br>(0.937)	−0.183<br>(0.157)	−0.195<br>(0.134)	0.029<br>(0.848)	−0.007<br>(0.966)

48	Right STG	0.064 (0.640)	0.122 (0.363)	0.122 (0.401)	0.223 (0.121)	−0.127 (0.387)	0.024 (0.866)	−0.013 (0.923)	−0.060 (0.645)	0.024 (0.878)	−0.123 (0.428)

*: $p < 0.05$; Note: the number outside the bracket was the standardized β value, and the number in the bracket was the *p*-value.

Table S5. The results of the multiple regression model on the nodal efficiency of each channel.

Channel	Brain Regions	Diagnosis		Age		Age²		Diagnosis : Age		Diagnosis : Age²	
		HbO	HbR	HbO	HbR	HbO	HbR	HbO	HbR	HbO	HbR
1	Left TPJ	0.049 (0.715)	−0.056 (0.680)	−0.212 (0.140)	−0.081 (0.574)	0.144 (0.319)	0.046 (0.749)	0.035 (0.789)	−0.137 (0.295)	0.084 (0.586)	−0.051 (0.744)
2	Left TPJ	0.082 (0.541)	−0.170 (0.207)	−0.199 (0.167)	−0.046 (0.749)	0.234 (0.107)	−0.175 (0.227)	0.008 (0.951)	0.038 (0.773)	−0.026 (0.865)	0.265 (0.089)
3	Left MFG	0.077 (0.547)	0.022 (0.865)	−0.294 (0.034) *	−0.191 (0.161)	0.143 (0.300)	−0.193 (0.160)	0.411 (0.001) *	0.145 (0.241)	−0.346 (0.021) *	−0.170 (0.249)
4	Left SFG	0.186 (0.164)	0.125 (0.338)	−0.221 (0.122)	−0.334 (0.017) *	0.045 (0.754)	−0.017 (0.903)	0.100 (0.438)	0.163 (0.197)	−0.207 (0.180)	−0.143 (0.343)
5	Left SMFG	0.085 (0.517)	0.224 (0.065)	−0.147 (0.293)	−0.398 (0.002) *	−0.107 (0.449)	−0.117 (0.365)	−0.075 (0.554)	0.185 (0.115)	0.092 (0.544)	−0.130 (0.351)
6	Right SMFG	0.105 (0.422)	0.189 (0.121)	−0.117 (0.402)	−0.432 (0.001) *	−0.117 (0.407)	−0.038 (0.772)	−0.053 (0.677)	0.099 (0.398)	−0.123 (0.415)	−0.131 (0.352)
7	Right SFG	0.176 (0.168)	0.156 (0.217)	−0.430 (0.002)	−0.460 (<0.001) **	0.193 (0.162)	0.082 (0.547)	0.077 (0.535)	0.199 (0.106)	−0.257 (0.083)	−0.022 (0.882)
8	Right MFG	0.014 (0.912)	−0.080 (0.547)	−0.122 (0.382)	−0.205 (0.149)	−0.129 (0.358)	−0.034 (0.810)	−0.057 (0.654)	0.201 (0.120)	−0.140 (0.353)	−0.116 (0.449)

9	Right TPJ	0.195 (0.140)	−0.273 (0.042)	−0.099 (0.479)	−0.131 (0.358)	−0.036 (0.800)	−0.074 (0.608)	0.124 (0.330)	0.031 (0.811)	−0.331 (0.031)	0.169 (0.275)
10	Right TPJ	0.059 (0.661)	−0.064 (0.621)	0.152 (0.295)	−0.312 (0.026) *	−0.190 (0.193)	0.036 (0.795)	−0.049 (0.711)	0.380 (0.003) *	0.075 (0.630)	−0.012 (0.938)
11	Left TPJ	0.049 (0.719)	−0.049 (0.718)	0.035 (0.807)	0.267 (0.066)	−0.157 (0.283)	−0.246 (0.091)	−0.026 (0.845)	−0.120 (0.359)	0.047 (0.763)	0.169 (0.280)
12	Left TPJ	−0.015 (0.910)	−0.100 (0.461)	−0.006 (0.968)	0.025 (0.861)	−0.014 (0.924)	−0.100 (0.494)	−0.061 (0.648)	0.115 (0.382)	−0.005 (0.976)	0.028 (0.859)
13	Left IFG	0.045 (0.741)	−0.048 (0.722)	0.018 (0.898)	0.042 (0.772)	−0.060 (0.681)	−0.064 (0.662)	−0.051 (0.701)	−0.121 (0.356)	0.134 (0.393)	0.211 (0.180)
14	Left MFG	0.080 (0.545)	0.016 (0.903)	−0.269 (0.059)	−0.158 (0.276)	0.082 (0.566)	−0.030 (0.837)	0.350 (0.007)	0.072 (0.585)	−0.134 (0.383)	0.029 (0.856)
15	Left SFG	−0.071 (0.586)	0.088 (0.491)	0.068 (0.627)	−0.167 (0.224)	−0.265 (0.062)	−0.096 (0.484)	−0.140 (0.271)	−0.137 (0.271)	−0.015 (0.921)	−0.037 (0.804)
16	Left SMFG	−0.032 (0.809)	−0.003 (0.980)	−0.182 (0.206)	−0.307 (0.025)	−0.046 (0.752)	−0.126 (0.357)	0.069 (0.595)	0.031 (0.802)	−0.06 (0.701)	−0.029 (0.844)
17	Right SFG	0.116 (0.383)	0.121 (0.345)	−0.238 (0.094)	−0.274 (0.047) *	0.081 (0.569)	−0.117 (0.395)	−0.014 (0.915)	0.057 (0.649)	0.018 (0.906)	−0.041 (0.782)
18	Right MFG	0.047 (0.716)	−0.184 (0.164)	−0.320 (0.023) *	−0.104 (0.459)	−0.018 (0.898)	−0.260 (0.068)	0.051 (0.687)	−0.002 (0.991)	0.012 (0.935)	0.285 (0.063)
19	Right IFG	0.004 (0.973)	0.064 (0.623)	0.057 (0.687)	−0.366 (0.010)	−0.275 (0.054)	0.214 (0.130)	−0.143 (0.265)	0.026 (0.839)	0.069 (0.651)	−0.026 (0.862)
20	Right TPJ	0.029 (0.827)	−0.083 (0.544)	−0.357 (0.013)	−0.041 (0.777)	0.097 (0.495)	−0.100 (0.495)	0.104 (0.416)	−0.031 (0.817)	−0.047 (0.761)	0.067 (0.67)
21	Right TPJ	0.152 (0.253)	−0.063 (0.642)	−0.286 (0.046) *	−0.017 (0.906)	0.037 (0.794)	−0.170 (0.245)	0.241 (0.064)	−0.015 (0.911)	−0.183 (0.234)	0.151 (0.336)

22	Right STG	−0.097 (0.476)	0.065 (0.629)	−0.153 (0.294)	0.224 (0.121)	0.050 (0.734)	−0.029 (0.842)	0.006 (0.964)	−0.042 (0.749)	0.066 (0.673)	−0.055 (0.725)
23	Left IFG	−0.098 (0.464)	−0.098 (0.455)	0.081 (0.570)	0.133 (0.344)	−0.064 (0.655)	0.057 (0.689)	−0.184 (0.157)	−0.318 (0.014)	0.292 (0.061)	0.210 (0.168)
24	Left MFG	−0.167 (0.211)	−0.123 (0.366)	−0.159 (0.265)	0.079 (0.585)	0.088 (0.540)	−0.120 (0.411)	−0.068 (0.600)	−0.188 (0.154)	0.213 (0.169)	0.209 (0.185)
25	Left SFG	−0.239 (0.077)	−0.048 (0.714)	0.042 (0.768)	−0.235 (0.092)	−0.204 (0.159)	−0.176 (0.210)	−0.030 (0.820)	0.233 (0.066)	0.138 (0.376)	0.021 (0.889)
26	Left SMFG	−0.180 (0.182)	−0.043 (0.746)	−0.084 (0.561)	−0.178 (0.211)	−0.125 (0.388)	−0.146 (0.306)	−0.037 (0.775)	0.080 (0.532)	0.134 (0.390)	0.177 (0.251)
27	Right SMFG	0.117 (0.382)	−0.076 (0.558)	0.115 (0.421)	−0.198 (0.155)	−0.168 (0.244)	−0.184 (0.190)	−0.078 (0.550)	0.127 (0.316)	−0.084 (0.587)	−0.037 (0.808)
28	Right SFG	−0.096 (0.476)	−0.158 (0.222)	−0.246 (0.088)	−0.181 (0.191)	0.003 (0.984)	−0.264 (0.059)	0.041 (0.750)	0.119 (0.343)	0.067 (0.665)	0.214 (0.156)
29	Right MFG	−0.233 (0.083)	−0.156 (0.250)	−0.014 (0.919)	0.142 (0.327)	−0.152 (0.292)	−0.166 (0.256)	−0.154 (0.237)	−0.029 (0.826)	0.161 (0.301)	0.087 (0.578)
30	Right IFG	−0.057 (0.672)	−0.002 (0.985)	0.077 (0.593)	0.351 (0.014)	−0.265 (0.069)	−0.343 (0.016)	0.053 (0.682)	−0.207 (0.106)	0.159 (0.308)	0.254 (0.097)
31	Right STG	0.084 (0.535)	−0.041 (0.766)	−0.184 (0.205)	0.027 (0.853)	0.063 (0.667)	−0.042 (0.777)	0.072 (0.586)	−0.017 (0.896)	−0.055 (0.728)	0.017 (0.914)
32	Left STG	0.080 (0.546)	0.085 (0.528)	−0.020 (0.887)	0.041 (0.777)	−0.135 (0.345)	0.125 (0.389)	0.097 (0.451)	0.054 (0.681)	0.173 (0.264)	−0.022 (0.887)
33	Left IFG	0.114 (0.398)	0.257 (0.051)	−0.041 (0.774)	0.052 (0.707)	−0.060 (0.679)	−0.195 (0.167)	0.065 (0.622)	0.057 (0.652)	0.070 (0.657)	0.005 (0.972)
34	Left MFG	−0.036 (0.789)	−0.066 (0.620)	−0.072 (0.623)	−0.14 (0.322)	−0.064 (0.663)	0.031 (0.828)	0.084 (0.525)	−0.190 (0.140)	0.045 (0.778)	0.123 (0.423)

35	Left SFG	−0.052<br>(0.686)	−0.094<br>(0.478)	−0.200<br>(0.149)	−0.034<br>(0.812)	0.004<br>(0.980)	−0.237<br>(0.098)	−0.177<br>(0.161)	−0.130<br>(0.315)	−0.022<br>(0.885)	0.221<br>(0.151)
36	Left SMFG	−0.025<br>(0.850)	−0.262<br>(0.051)	0.071<br>(0.622)	0.001<br>(0.995)	−0.202<br>(0.165)	−0.227<br>(0.116)	0.112<br>(0.391)	−0.094<br>(0.468)	0.061<br>(0.694)	0.231<br>(0.138)
37	Right SFG	−0.213<br>(0.100)	−0.228<br>(0.086)	−0.235<br>(0.090)	−0.217<br>(0.125)	0.125<br>(0.367)	0.066<br>(0.643)	−0.192<br>(0.127)	−0.054<br>(0.671)	0.057<br>(0.702)	0.235<br>(0.126)
38	Right MFG	0.023<br>(0.863)	0.015<br>(0.913)	−0.107<br>(0.461)	−0.037<br>(0.796)	0.119<br>(0.419)	−0.227<br>(0.113)	0.083<br>(0.532)	−0.071<br>(0.580)	−0.139<br>(0.380)	0.044<br>(0.772)
39	Right IFG	−0.045<br>(0.742)	0.032<br>(0.813)	0.058<br>(0.689)	0.223<br>(0.123)	−0.156<br>(0.286)	−0.138<br>(0.341)	−0.070<br>(0.597)	−0.186<br>(0.157)	0.184<br>(0.243)	0.105<br>(0.500)
40	Right STG	0.089<br>(0.505)	−0.006<br>(0.963)	0.235<br>(0.101)	0.320<br>(0.026)	−0.178<br>(0.217)	−0.158<br>(0.271)	−0.013<br>(0.921)	−0.136<br>(0.296)	0.101<br>(0.516)	0.160<br>(0.303)
41	Left STG	0.058<br>(0.663)	0.238<br>(0.074)	0.143<br>(0.312)	−0.127<br>(0.370)	−0.256<br>(0.074)	−0.067<br>(0.639)	0.133<br>(0.302)	0.169<br>(0.192)	0.087<br>(0.571)	−0.093<br>(0.544)
42	Left IFG	0.130<br>(0.324)	0.262<br>(0.045) *	0.262<br>(0.065)	0.150<br>(0.279)	−0.255<br>(0.074)	−0.226<br>(0.107)	−0.126<br>(0.326)	0.135<br>(0.283)	0.129<br>(0.398)	−0.150<br>(0.320)
43	Left SFG	−0.180<br>(0.174)	−0.029<br>(0.827)	0.031<br>(0.824)	−0.024<br>(0.864)	−0.049<br>(0.727)	−0.150<br>(0.290)	−0.278<br>(0.031)	−0.101<br>(0.429)	0.028<br>(0.852)	−0.102<br>(0.505)
44	Left SMFG	−0.269<br>(0.042)	0.052<br>(0.696)	0.203<br>(0.150)	−0.296<br>(0.038)	0.015<br>(0.916)	0.001<br>(0.994)	−0.260<br>(0.043)	0.206<br>(0.112)	0.241<br>(0.115)	−0.117<br>(0.445)
45	Right SMFG	−0.084<br>(0.536)	0.125<br>(0.357)	0.098<br>(0.497)	0.047<br>(0.746)	−0.084<br>(0.564)	−0.045<br>(0.756)	0.033<br>(0.805)	−0.074<br>(0.574)	−0.015<br>(0.924)	0.032<br>(0.841)
46	Right SFG	0.028<br>(0.834)	0.018<br>(0.896)	0.102<br>(0.476)	−0.027<br>(0.853)	−0.115<br>(0.426)	−0.141<br>(0.333)	−0.200<br>(0.126)	0.011<br>(0.931)	0.161<br>(0.301)	−0.043<br>(0.785)
47	Right MFG	−0.082<br>(0.544)	0.010<br>(0.939)	0.213<br>(0.143)	0.157<br>(0.278)	−0.118<br>(0.417)	−0.109<br>(0.455)	−0.130<br>(0.323)	−0.179<br>(0.176)	0.022<br>(0.890)	0.110<br>(0.485)

48	Right STG	0.041 (0.756)	0.103 (0.449)	−0.059 (0.678)	0.137 (0.344)	−0.208 (0.149)	−0.158 (0.278)	0.053 (0.682)	−0.011 (0.935)	0.115 (0.457)	−0.026 (0.868)

*: $p < 0.05$, **: $p < 0.001$, Note: the number outside the bracket was the standardized β value, and the number in the bracket was the p-value.
